# Training on involving cognitions and perceptions in the occupational health management and work disability assessment of workers: development and evaluation

**DOI:** 10.1186/s12909-021-03084-x

**Published:** 2022-01-07

**Authors:** Mariska de Wit, Nina Zipfel, Bedra Horreh, Carel T. J. Hulshof, Haije Wind, Angela G. E. M. de Boer

**Affiliations:** Department of Public and Occupational Health, Coronel Institute of Occupational Health, Amsterdam Public Health Research Institute, Amsterdam UMC, University of Amsterdam, PO Box 22700, 1100 DE Amsterdam, The Netherlands

**Keywords:** Occupational health, Occupational physicians, Insurance physicians, Cognition, Perception

## Abstract

**Background:**

In order to improve work participation of workers with a chronic disease, it is important for occupational health professionals (OHPs) to focus on those factors that can influence work participation. Cognitions and perceptions, such as recovery expectations and self-efficacy, are examples of these factors that can influence work participation. However, no training program is available for OHPs on how to involve cognitions and perceptions during their practice. Therefore, the aim of this study was to develop a training program for OHPs on how to involve cognitions and perceptions in the occupational health management and work disability assessment of workers with a chronic disease. In addition, to evaluate the OHPs’ satisfaction with the training and the feasibility of the training and learned skills.

**Methods:**

The training program was developed using information from previously conducted studies regarding cognitions and perceptions in relation to work participation. Satisfaction with the training by OHPs was evaluated by means of a questionnaire. A smaller group of OHPs were interviewed three to six months after the training to evaluate the feasibility of the training and learned skills.

**Results:**

The 4.5-h training program consisted of four parts concerning: 1) cognitions and perceptions associated with work participation, 2) how to obtain information on them, 3) the course of the conversation on these factors, and 4) intervening on these factors. Eight training sessions were conducted with 57 OHPs, of whom 54 evaluated the training. Participants were very satisfied (score 8.5 on a scale from 1 to 10). The eleven interviewed participants were more aware of cognitions and perceptions during consultations and perceived the training to be feasible. However, not all participants had applied the acquired skills in their practice, partially because of a lack of time.

**Conclusions:**

OHPs are very satisfied with the training program and perceive it to be feasible. The training increases awareness of important cognitions and perceptions and may possibly help to increase work participation of workers with a chronic disease.

**Supplementary Information:**

The online version contains supplementary material available at 10.1186/s12909-021-03084-x.

## Background

A chronic disease can limit physical and mental functioning, which can have a negative impact on work participation [1, 2]. The World Health Organization defines a chronic disease as a disease with a long duration and generally slow progression [3]. In order to improve work participation of workers with a chronic disease, it is important for occupational health professionals (OHPs), who make important decisions concerning work participation of workers with health problems, to focus on those factors that can influence work participation. Cognitions and perceptions, such as recovery expectations and self-efficacy, are examples of these factors that can influence work participation [4, 5]. Evidence was found for an association between ten different cognitions and perceptions and work participation: recovery and return to work (RTW) expectations, self-efficacy, feelings of control, perceived health, fear-avoidance beliefs, perceived work-relatedness, coping strategies, catastrophizing, motivation and optimism/pessimism [6].

In general, OHPs recognize the importance of such cognitions and perceptions [7–9]. In a study by Achterberg et al. [7] motivation, coping strategies, own expectations for work participation and self-perceived health were identified by insurance physicians (IPs) and labor experts as factors that are important for work participation in young disabled persons in the Netherlands. In a study by Peters et al. [9] experts on work disability agreed that motivation to RTW, self-efficacy, positive coping skills, having no catastrophizing thoughts and having no fear-avoidance beliefs were important facilitators for RTW following surgery.

However, obtaining reliable information on these cognitions and perceptions from workers themselves is a challenge. In our earlier studies, the majority of the OHPs and workers agreed that information on these factors should be obtained during consultations with workers [10, 11]. Whether information about cognitions and perceptions can be obtained during consultations depends on the course of these conversations and the disclosure by workers. Previous studies have shown that factors such as a lack of trust and lack of listening can have a negative influence on disclosure during consultation, which makes it difficult for OHPs to obtain reliable information [12, 13].

If OHPs succeed in obtaining information on cognitions and perceptions, the second challenge is to try to influence these factors if they are hindering work participation. In this regard, OHPs need to be aware of existing effective interventions. Sullivan et al. [14] described the Progressive Goal Attainment Program that was able to reduce catastrophizing thoughts – a predictor of RTW. Leensen et al. [15] described a multidisciplinary rehabilitation program that increased the self-efficacy of workers with cancer and had a positive influence on RTW.

Although it is important for OHPs to know how to obtain information on workers’ cognitions and perceptions and how to intervene on these factors, currently no specific training exists for OHPs. Therefore, the purpose of the present study was: 1) to develop a training program for OHPs on involving workers’ cognitions and perceptions in the occupational health management and work disability assessment of workers, 2) to evaluate the OHPs’ satisfaction with the training, and 3) to evaluate the feasibility of the training and learned skills three to six months after the training.

## Methods

The Medical Ethics Review Committee of the Academic Medical Center (AMC), University of Amsterdam, confirmed that the Medical Research Involving Human Subjects Act (WMO) does not apply to this study and that official approval by this committee was therefore not required (W 19__174 # 19.213 and W 19_494 # 20.012).

### Participants

The OHPs who participated in the training were occupational physicians (OPs), OPs in training, IPs and IPs in training, working in the Netherlands. The main task for OPs in the Netherlands is prevention of work-related diseases, promoting health, and occupational health management of workers with health problems. IPs aim to increase work participation by evaluating the functional abilities of workers and by determining whether workers should receive a work disability benefit. To become an OP or IP in the Netherlands, physicians in training must complete a postgraduate program of four years during which they gain a lot of practical experience. To maintain being registered as a specialist every five years, OPs and IPs need to participate in accredited activities, such as continuing medical education, and in inter-collegial peer review.

### Procedure

#### Step 1. Development of the training

The main goals of the training program for OHPs are acquiring knowledge of cognitions and perceptions associated with work participation, identifying these cognitions and perceptions, and changing these cognitions and perceptions when necessary. The information provided during the training was based on four previously conducted studies [6, 10, 11, 16]. Results of a systematic review formed the basis for the content about the cognitions and perceptions important for work participation [6]. Information concerning methods physicians can apply to obtain information in order to identify cognitions and perceptions was retrieved from results of a survey study among physicians [10]. Information about factors that may influence the course of a conversation concerning cognitions and perceptions was retrieved from a focus group study among workers with chronic health problems [11]. A scoping review about interventions that are aimed at changing cognitions and perceptions and improving work participation formed the basis for the final part of the training program, in combination with information on how to change cognitions and perceptions retrieved from different guidelines for OPs and IPs [16].

Various exercises were developed for practicing with the learned information and acquired skills during the training. By basing the exercises on real client cases provided by OHPs and developing exercises in which participants needed to discuss their own client cases with each other, a clear connection was made between theory and practice.

In order to help OHPs to apply the learned skills in daily practice, a conversation tool was developed for them. The first version of this conversation tool was tested on face validity by a patient representative, three IPs and two OPs who were not participants in the training. The conversation tool was adapted on the basis of their feedback, collected during phone interviews.

#### Step 2. Evaluation of OHPs’ satisfaction with the training

In October and November 2019, eight training sessions were scheduled for the OPs and IPs. OPs and IPs who were interested in participating received an email with information about the training program and signed an informed consent form.

After the training the participants received a questionnaire on paper about their satisfaction in respect of different aspects of the training, which they could complete immediately or at home. The anonymous questionnaire consisted of ten statements about reaching the most important goals of the training (e.g. knowing important cognitions and perceptions, identifying them and changing them when necessary) that had to be scored on a 5-point Likert scale (completely disagree – completely agree) and eight statements about the design of the training program (e.g. duration, level, content) that had to be scored on a 5-point Likert scale (very dissatisfied – very satisfied), with room for explanation. Besides this, they had to rate their satisfaction with the complete training on a scale from 1 to 10 (very dissatisfied – very satisfied). There were five “yes” or “no” questions concerning the possible implementation of the conversation tool and learned skills in practice and four open questions about positive and negative elements of the training and barriers to and facilitators for implementation. Finally, there was room for additional comments.

#### Step 3. Interviews concerning feasibility of the training and learned skills

In January 2020, the OPs and IPs who were willing to participate in an interview study concerning the feasibility of the training and learned skills in practice were recruited by e-mail, and written consent was obtained. The structured interviews were conducted by the researchers by phone, audio-recorded and transcribed. The framework for the interview guide consisted of the following feasibility aspects proposed by Bowen et al. [17]: acceptability, demand, implementation, practicality, adaptation, integration and limited-efficacy testing. The aim of this feasibility study was to reflect on the experience of the participants with the training and with the learned skills in practice. An analysis concerning practical aspects on future implementation, which were addressed in the questions concerning the aspects practicality, integration and factors affecting implementation ease or difficulty, will be reported in a separate study.

### Data analysis

Statistical data was analyzed using SPSS statistics 26.0. Descriptive statistics were used to describe the scores on the questionnaires and to describe the characteristics of the participants who participated in the training program and in the interviews. The answers to the open questions from the questionnaires were summarized by one researcher (MdW) and checked by the other researchers (BH, AdB, HW, CH).

The transcripts of the interviews were coded using MAXQDA 2020 Software [18]. For the coding, a mixed concept-driven and data-driven approach was applied and the steps described by Kuckartz et al. [19] were followed. The starting point and main categories from the coding system were the feasibility aspects [17]. Subsequently, sub-codes were assigned using a data-driven approach. All interviews were coded independently by two researchers (MdW and NZ). Afterwards, the codes were discussed by the two researchers until consensus was reached. Quotations were presented to illustrate the findings.

## Results

### Content of the training

The evidence-based training program has a duration of 4.5 h (including breaks) and consists of four main parts, which are described in Table 1. During the training the participants participate in different exercises, which are all discussed in class afterwards. During the training, a conversation tool is presented to the participants to help them to apply the learned skills in daily practice. The tool can be used before, during and/or after consultations with workers with a chronic disease. The first page of the tool consists of a list of the cognitions and perceptions with their definitions and instructions about how to use the tool. The second and third page of the tool contain questions which OHPs could ask to obtain information concerning all ten different cognitions and perceptions. There is also space for additional questions OHPs could write down themselves. Next to these questions is a checklist in which OHPs can indicate whether or not a cognition or perception has a limiting effect on work participation for the worker in question. The final page of the conversations tool consists of an overview of indicators for limiting or promoting cognitions and perceptions, so that OHPs can assess the presence of these cognitions and perceptions. Indicators for limiting cognitions and perceptions are presented in red and indicators for promoting cognitions and perceptions are presented in green.

**Ta﻿ble 1:** Overview of training on cognitions and perceptions

Module	Time	Content
Introduction	15 min	• General introduction about the learning goals of the training • Introduction round to get to know each other
Part 1: Importance of cognitions and perceptions	45 min	• Information about ten cognitions and perceptions associated with work participation: recovery and return to work expectations, self-efficacy, feelings of control, perceived health, fear-avoidance beliefs, perceived work-relatedness, coping strategies, catastrophizing, motivation and optimism/pessimism • Exercise in which participants discuss in pairs which cognitions and perceptions they think are most important • Information about importance of cognitions and perceptions according to OPs and IPs in the Netherlands
Part 2: Obtaining information	60 min	• Information about how to obtain information concerning cognitions and perceptions • Exercise in which participants in pairs identify cognitions and perceptions in four different written cases • Exercise in which participants in pairs think up and write down questions that could be asked to obtain information regarding all ten cognitions and perceptions • Explanation about the conversation tool
Part 3: Course of the conversation	45 min	• Exercise in which all participants are divided into two groups. One group needs to mention as many factors that can positively influence the course of the conversation as possible. The other group needs to mention as many factors as possible that can negatively influence the course of the conversation. The group which mentions the most correct factors, wins • Information about factors that can influence the course of the conversation according to workers with a chronic disease
Part 4: Interventions	55 min	• Exercise in which participants write down how they dealt with workers with limiting cognitions and perceptions in the past and discuss this in small groups • Information about methods to change cognitions and perceptions, such as cognitive behavioral therapy, graded exposure or motivational interviewing
Debriefing	20 min	• Discussion about the most important things learned during the training according to the participants

*OPs* Occupational physicians, *IPs* Insurance physicians

All information concerning the training is bundled in a trainers’ manual and a PowerPoint which is presented during the training. Eligible trainers are OPs and IPs with at least five years of experience in occupational health care. The experienced OPs and IPs can be a role model for the participants, which can support implementation of the learned skills into practice [20].

### OHPs’ satisfaction with the training

In total 57 physicians participated in the training program, among which 24 OPs, 6 OPs in training, 17 IPs and 10 IPs in training. The participants had a mean age of 51.2 years (SD = 11.9) and 26 of the participants were women. The participants had an average of 17.3 years (SD = 12.0) work experience in their current function. In total 54 of the 57 OPs and IPs who participated in the training program completed the anonymous questionnaire concerning their satisfaction with the training (response rate: 94.7%).

Overall, the respondents of the questionnaire were very satisfied with the training. Of the total number of participants, 52 respondents gave a mean score of 8.5 (SD = .8) on a scale from 1 to 10 for their overall satisfaction with the training.

More than 90% of the respondents “agreed” or “completely agreed” that 9 of the 10 goals of the training had been met (Table 2). In contrast, only 77% indicated that they had reached the goal concerning knowing about interventions focused on cognitions and perceptions and improving work participation.

**Table 2:** Scores on 5-point Likert scale (completely disagree – completely agree) on statements about reaching the goals of the training

Statements	N	Percentage of participants with score 4 (agree) or 5 (completely agree)	Minimum and maximum scores
1. I know important cognitions and perceptions	53	98	3–5
2. I know the ten cognitions and perceptions associated with work participation	52	98	3–5
3. I know how information can be obtained	53	94	3–5
4. I know which questions to ask to obtain information	53	92	3–5
5. I know the conversation tool and how it can be used	53	94	2–5
6. I know indicators for cognitions and perceptions	53	94	3–5
7. I am able to recognize cognitions and perceptions	52	96	3–5
8. I know the prerequisites for having a conversation	52	92	2–5
9. I know the positive and negative influences on the course of a conversation	53	91	2–5
10. I know which interventions can support work participation	53	77	3–5

In Table 3 scores are presented regarding satisfaction with the training. All respondents who scored the statements were “satisfied” or “very satisfied” about the assignments, the explanation in class, the cases that were presented and the appendices that were handed out. One person was dissatisfied with the group size of the training and three respondents were dissatisfied with the duration of the training.

**Table 3:** Scores on 5-point Likert scale (very dissatisfied – very satisfied) on statements about the design of the training

Statements	N	Percentage of participants with score 4 (satisfied) or 5 (very satisfied)	Minimum and maximum scores
How satisfied are you with…			
1. the level of the training	54	98	3–5
2. the assignments during the training	53	100	4–5
3. the explanation in class during the training	53	100	4–5
4. the cases that were presented during the training	53	100	4–5
5. the group size of the training	53	96	2–5
6. the duration of the training	53	91	2–5
7. the appendices that were handed out during the training	52	100	4–5
8. the conversation tool	52	98	3–5

Overall, 51 out of 52 respondents to the questionnaire agreed that the training was useful for practice. Fifty-two out of 53 respondents expressed the intention to use the learned skills and the appendices, and 50 out of 52 respondents had the intention to apply the conversation tool in practice. Reasons for not doing so were a lack of experience and not seeing workers during consultations more than once. Forty-eight out of 49 respondents thought that the training could be implemented in practice and 51 out of 52 respondents would recommend the training program to their colleagues.

In the open-ended questions participants mentioned positive and negative components of the training. Overall, respondents appreciated the interaction during the training, the assignments and the debriefing after the assignments. However, some respondents reported that there was not enough time to practice the learned skills during the training, in particular for the final part of the training program, concerning interventions.

Respondents indicated that the implementation of the training into practice would be facilitated if the training was implemented in the standard education for physicians or if awareness was raised about the existence of the training program in professional associations or trade journals. Time was perceived to be the most important barrier for implementation.

### Feasibility of the training and learned skills

Eleven participants were interviewed regarding the feasibility of the training and learned skills, and saturation was reached. Among the participants were five OPs (of whom one in training) and six IPs (of whom two in training). These participants included five females and six males, with an mean age of 48.5 (SD = 12.9) years and 15.4 (SD = 14.2) years of work experience. The time between the training and the interview varied between 13 and 25 weeks. The codes and sub-codes that were assigned to the interviews with corresponding quotations are presented in the table in Additional file 1.

#### Acceptability

In general, the participants were very satisfied with the training. It was perceived to be entertaining, clear and informative and its topic was relevant. Participants appreciated the fact that OPs and IPs were mixed in the training:

*“[…] a lot of our work is similar and I think that it is useful to have both parties present during the training, because you also learn about each other’s perceptions from each other’s cases […]”(P6)*

Most participants thought the content of the training was appropriate for their practice and that no changes were necessary. However, one participant felt that the training should be less focused on recommending interventions, but more focused on how to adapt conversation techniques to limiting cognitions and perceptions. Another participant mentioned that the training did not completely fit within his organization because, due to work pressure, the focus there was more on assessments of the functional abilities of workers than on guiding them back to work.

Overall, participants were satisfied with the tool for facilitating the use of the learned skills in practice. They thought the tool was clear and they were satisfied with its questions and arrangement. Some participants thought the tool was inappropriate to use during consultation. They said it was inconvenient or even embarrassing for physicians to look at the tool during their conversations:

*“Well I also find it a bit embarrassing to be looking at a page like this during a consultation […] I should be able to do it by heart.”(P3)*

They also indicated that workers might disagree with the physician’s thoughts about the cognitions and perceptions, which could make it inappropriate to talk about them. One participants doubted if all her colleagues were open to this conversation tool.

#### Demand

Overall, the participants agreed that the training is valuable for physicians. Participants mentioned that there was room for improvement in the practice of physicians when it comes to this topic. Especially, they appreciated the fact that the training was based on scientific evidence. However, some participants mentioned that physicians implicitly already take some perceptions into account. One of the participants said the training was not useful in practice, because the training on its own would not fit into the education of the physicians.

Most of the participants mentioned that the learned skills and tool were valuable and that they used it in practice. Participants tried to identify cognitions and perceptions, used the indicators and discussed the cognitions and perceptions more during consultations than before:

*“[…] to be honest I never did it (considered cognitions and perceptions during consultations), or only if it was so clear that it really stood out, but now I try to do it systematically, so for every client.”(P4)*

Some participants only implicitly used the learned skills. According to most participants, the learned skills can be used for consultations with all clients, although one participant used it especially when clients had psychological complaints.

However, participants did not always use the tool during consultations. Some of the participants said that they looked at the tool before consultations, in order to prepare for the consultations, or after consultations. There were also participants who did not use the tool or all learned skills, did not change the interventions they recommend or did not change anything in their reports. However, participants were still motivated to pay more attention to what they had learned and to use the skills. They planned to use the tool in scheduled consultations or planned to internalize or practice more with the cognitions and perceptions and questions they can use to obtain information, so they did not have to constantly look at the tool during consultations.

#### Implementation

Although the participants had the time and opportunity to participate, they said OPs and especially IPs are very busy, so time would be a main barrier to implementation of the training.

In respect of the implementation of the learned skills, time was also perceived to be a barrier. Participants mentioned they lacked time during consultations because a lot of information needs to be discussed. Some mentioned that they did not have enough time to acquaint themselves better with the tool in order to use it. However, there were participants who reported having enough time and room to use the learned skills and tool. One participant even mentioned that, because of the training, he saved time during consultations, since recognizing cognitions and perceptions was easier than before.

Another barrier for implementing the learned skills was that cognitions and perceptions were sometimes not easy to recognize. In the cases discussed during the training cognitions and perceptions were more obvious, while in practice they were sometimes less clear:

*“What I particularly noticed was that you can’t just quickly identify a certain cognition. […] it’s often a lot more subtle. It takes a bit more effort to discover what it is.”(P4)*

#### Adaptation

The majority of the physicians agreed that the training was suitable for OPs and IPs, because both professionals see workers with chronic diseases who experience limiting cognitions and perceptions. However, some participants felt that the training was more suitable for OPs than for IPs, because OPs see workers multiple times, while sometimes IPs only see them once and OPs recommend interventions to workers more often than IPs.

#### Limited efficacy

Participants mentioned that the training was a kind of eye-opener to the importance of cognitions and perceptions. The training increased their awareness of cognitions and perceptions during consultations. Participants had the feeling that the cognitions and perceptions were better outlined than before and that they were better at recognizing and naming them:

*“During the training I found it a real eye-opener that, oh, you can use these terms, and you can ask these questions.”(P1)*

Information about the cognitions and perceptions helped to form a complete picture and predict the future ability of the client and can be used as input for following consultations with the worker. However, some participants did not have the feeling that the training led to changes in the work disability assessment.

## Discussion

### Key findings

A four-part training program was developed for OHPs to acquire knowledge of cognitions and perceptions important to work participation of workers with a chronic disease, how to obtain information concerning these factors, the course of the conversation on these factors and intervening on cognitions and perceptions. OHPs who participated in the training were very satisfied overall with the design and content of the training. Directly after the training the participants felt that their knowledge had been improved. They were motivated and intended to use the learned skills with the corresponding conversation tool in practice. Three to six months after the training, the participants indicated being more aware of cognitions and perceptions during consultations and having the feeling they were better outlined than before. Although the training was perceived feasible, not all participants used all the learned skills and the corresponding tool during consultations.

The training was perceived important and suitable for both OPs and IPs, partly because participants see people with the same kind of cognitions and perceptions. This was also found in previous studies, the results of which showed that both OPs and IPs agree that multiple cognitions and perceptions are important for work participation [6, 7, 21]. The perceived importance of cognitions and perceptions for work participation is also an explanation for why almost all OPs and IPs were planning to apply the learned skills and conversation tool in practice. The developed training program contained different components that might help improve the effectiveness of training in changing the behavior of physicians as mentioned in previous studies by Mostofian et al. [22] and Berkhof et al. [23] The training was active and provided room for discussion between the physicians, and during the training a clear link was made between what the physicians learned and actual cases from their practice.

However, three to six months after they had done the training the eleven participants interviewed mentioned that they did not use everything they had learned. Participants mentioned three main problems for implementing the learned skills in practice. The first problem is that sometimes cognitions and perceptions are still hard to identify during consultations. Although during training the participants had the opportunity to practice the skill of identifying cognitions and perceptions in different cases, this practice might not reflect real-life cases enough because they were written cases. Results of a review by Berkhof et al. [23] concerning teaching communication skills to physicians indicate that role-play with actors might be more effective in helping the physicians to practice this skill in the future than exercises with written cases. The second problem with the implementation is that looking at the conversation tool during consultations was perceived to be inappropriate. Possibly, looking at the tool disrupts eye contact between the worker and the physician and can give the worker the feeling that the physician is not well prepared. Both aspects are important for the course of the conversation between physician and client [24, 25]. A solution for OPs and IPs might be to internalize the cognitions, perceptions and corresponding questions, which many of the interviewed participants were already planning to do. However, this automatically leads to third problem for implementation: a lack of time, which can form an obstruction to OPs and IPs internalizing the cognitions and perceptions. This was also perceived to be a barrier in previous studies about implementing new skills for OPs and IPs in practice [26, 27].

The reasons mentioned for not using all learned skills raise the question whether a 4.5-h training program is long or comprehensive enough to learn OHPs how to involve cognitions and perceptions during practice. According to the review by Berkhof et al. [23], training programs on communication skills for physicians should have a duration of at least one day. Although the short duration of the training makes participating in the training for OHPs feasible, extra time during the training or a follow-up training program might possibly improve the feasibility of applying the learned skills in practice. This might give OHPs more time to practice identifying cognitions and perceptions and internalizing the cognitions and perceptions, which may remove the need for OHPs to look at the tool, and save time before and during consultations. Besides, extra time during the training or a follow-up training could give OHPs more time to learn and discuss interventions focused on cognitions and perceptions and improving work participation, because not every OHP had the feeling that they knew the interventions directly after the training.

In addition to extending the duration of the training and offering follow-up training, offering the training at an early stage in the education of OHPs might improve the feasibility of the learned skills. Implementing the acquired skills during practice may be easier for OHPs in training than for experienced physicians, because for experienced physicians this often involves altering long-established practices [28].

### Strengths and limitations

A strength of this study is that the developed training program is based on evidence from previously conducted studies. The exercises included in the training are based on real-life cases from OHPs, which may bridge the gap between theory and practice. The new training program was extensively evaluated by means of questionnaires and in-depth interviews.

A limitation of this study is the fact that, in the satisfaction questionnaires directly after the training, we did not ask whether participants were OPs, IPs, or in training and therefore, we were not able to analyze whether OPs and IPs evaluated the training differently. This might have been relevant because some of the interviewees said this training is more suitable for OPs than for IPs. Another limitation is that not all participants responded to all questions in the questionnaire and we did not assess the reasons for not responding. In addition, not all participants were available to be interviewed at follow-up. It is possible that the participants who wanted to be interviewed were, in general, more positive about their experiences with putting the learned skills into practice.

### Implications for practice and future research

The developed training program can help OHPs to identify important cognitions and perceptions during consultations and to know how to intervene on these cognitions and perceptions in order to improve work participation. After the training, participants mentioned being better equipped to recognize cognitions and perceptions during consultations. However, to know whether the training truly has an effect on the ability to recognize cognitions and perceptions during consultations, a further study with a robust design, i.e. a randomized controlled trial, should be conducted. Besides, study is required into whether this training has an effect on the work participation of the workers with a chronic disease. Studies should also be conducted to test whether the learned information and skills are also applicable in consultations with workers who have non-chronic health problems. Finally, study is also required into whether increasing the length of the training, adding follow-up training or offering the training at an early stage in education of OHPs could improve the feasibility of the learned skills in practice.

## Conclusions

In this study, a training program was developed which may help OHPs to involve workers’ cognitions and perceptions in the occupational health management and work disability assessment of these workers. OHPs were very satisfied with the training and perceived it to be feasible, although not all OHPs used all learned skills in practice. After the training, OHPs mentioned they were more aware of the cognitions and perceptions and they were better equipped to recognize them during consultations, which can help them in their efforts to improve work participation in workers with a chronic disease.

## Supplementary Information

**Additional file 1: Table 1**. Code system of interviews.

## Data Availability

The anonymized data used and/or analysed during the current study are available from the corresponding author on reasonable request.

## Abbreviations

AMC: Academic Medical Center
IPs: Insurance physicians
OHPs: Occupational Health Professionals
OPs: Occupational physicians
RTW: Return to work
WMO: Medical Research Involving Human Subjects Act
