# Supplementary material for: Training on involving cognitions and perceptions in the occupational health management and work disability assessment of workers: development and evaluation
Source: BMC Med Educ. 2022 Jan 7;22:20. doi: 10.1186/s12909-021-03084-x (PMC8740490; doi:10.1186/s12909-021-03084-x)
Supplement: Supplementary file 1 — Additional file 1: Table 1. Code system of interviews. [file 12909_2021_3084_MOESM1_ESM.docx]

**Training on involving cognitions and perceptions in the occupational health management and work disability assessment of workers: development and evaluation**

Mariska de Wit, PhD, Nina Zipfel, PhD, Bedra Horreh, MSc, Carel T. J. Hulshof, MD, PhD, Haije Wind, MD, PhD, Angela G.E.M. de Boer, PhD

Amsterdam UMC, University of Amsterdam, Department of Public and Occupational Health, Coronel Institute of Occupational Health, Amsterdam Public Health research institute, Amsterdam, The Netherlands

e-mail address corresponding author: m.e.dewit@amsterdamumc.nl

**Additional file 1.**

Table 1. Code system of interviews three to six months after the training (N = 11)

| **Feasibility aspects Bowen** | **Sub-themes** | **Data-driven sub-codes** |
| --- | --- | --- |
| **Acceptability** | *Satisfaction* | - Satisfied with topic |
|  |  | - Satisfied with training |
|  |  | - Satisfied with mix OPs and IPs |
|  |  | - Satisfied with tool |
|  |  | - Not satisfied with tool |
|  | *Perceived appropriateness* | - No changes necessary |
|  |  | - Less focus on interventions during training |
|  |  | - Not appropriate during consultation |
|  |  | - Not suitable to apply to all clients |
|  | *Fit within organizational culture* | - Organizational readiness |
|  |  | - Organizational support |
| **Demand** | *Perceived demand* | - Valuable |
|  |  | - Confidence in own ability |
|  |  | - Not useful |
|  | *Actual use* | - Used |
|  |  | - Not used |
|  | *Expressed interest or intention to use* | - Intrinsic motivation |
|  |  | - Intend to use |
|  |  | - Internalizing |
| **Implementation** | *Success or failure of execution* | - No barriers to application |
|  |  | - Difficult to recognize factors |
|  | *Amount, type of resources needed to implement* | - Enough time during consultation |
|  |  | - Enough time during preparation of consultation |
|  |  | - Lack of time during consultation |
|  |  | - Lack of time in general |
| **Adaptation** | *Population adaptation* | - Suitable for OPs and IPs |
|  |  | - Not suitable for IPs |
|  |  | - Suitable for OPs |
| **Limited efficacy** | *Intended effects of program or process on key variables* | - Eye opener/increased awareness |
|  |  | - Identify more accurately |
|  |  | - Effect on assessment |
|  |  | - Input for consultation |
|  |  | - No effect on assessment |

*OPs* Occupational physicians, *IPs* Insurance physicians
